# Residue contact-count potentials are as effective as residue-residue contact-type potentials for ranking protein decoys

**DOI:** 10.1186/1472-6807-8-53

**Published:** 2008-12-08

**Authors:** Dan M Bolser, Ioannis Filippis, Henning Stehr, Jose Duarte, Michael Lappe

**Affiliations:** 1 The Max Planck Institute for Molecular Genetics, Berlin, Germany

## Abstract

**Background:**

For over 30 years potentials of mean force have been used to evaluate the relative energy of protein structures. The most commonly used potentials define the energy of residue-residue interactions and are derived from the empirical analysis of the known protein structures. However, single-body residue 'environment' potentials, although widely used in protein structure analysis, have not been rigorously compared to these classical two-body residue-residue interaction potentials. Here we do not try to combine the two different types of residue interaction potential, but rather to assess their independent contribution to scoring protein structures.

**Results:**

A data set of nearly three thousand monomers was used to compare pairwise residue-residue 'contact-type' propensities to single-body residue 'contact-count' propensities. Using a large and standard set of protein decoys we performed an in-depth comparison of these two types of residue interaction propensities. The scores derived from the contact-type and contact-count propensities were assessed using two different performance metrics and were compared using 90 different definitions of residue-residue contact. Our findings show that both types of score perform equally well on the task of discriminating between near-native protein decoys. However, in a statistical sense, the contact-count based scores were found to carry more information than the contact-type based scores.

**Conclusion:**

Our analysis has shown that the performance of either type of score is very similar on a range of different decoys. This similarity suggests a common underlying biophysical principle for both types of residue interaction propensity. However, several features of the contact-count based propensity suggests that it should be used in preference to the contact-type based propensity. Specifically, it has been shown that contact-counts can be predicted from sequence information alone. In addition, the use of a single-body term allows for efficient alignment strategies using dynamic programming, which is useful for fold recognition, for example. These facts, combined with the relative simplicity of the contact-count propensity, suggests that contact-counts should be studied in more detail in the future.

## Background

Accurate descriptions of the different non-covalent interactions involved in protein folding and stability are essential for a number of related problems. Potential energy functions based on such terms have been widely used to facilitate: fold recognition [1-3], homology modelling [4,5], docking [6], *ab-initio *structure prediction [7-9], sequence design [10] and the analysis of protein folding kinetics [11,12]. In each case, the purpose of the potential function is to discriminate between a variety of alternative conformations, selecting the most energetically favourable (assumed to be the most native) for further analysis [13]. Different potential energy functions have been defined at different levels of structural resolution [14]. At the atomic level, various pairwise inter-atom potentials (force-fields) have been developed from the detailed analysis of small, protein-like compounds. These include: ECEPP [15,16], MM [17,18], AMBER [19,20], CHARMM [21-23] and GROMOS [24]. Potential functions between distinct groups of atoms have also been defined, typically between pairs of residues [8,25-28] or idealised elements of secondary structure [9,29-34]. These 'potentials of mean force' (mean-fields) have the nature of free energies [27,35], and may be derived by conformational averaging [7] or, more commonly, by empirical methods as described below.

There are two commonly used methods for deriving empirical potential energy functions [36]. The first method employs a statistical analysis of the observed 'interactions' [8,25,26,37,38]. In this method, the observed occurrence of a particular interaction is weighted by its expected occurrence in a given reference state [27,39]. The resulting statistical interaction propensities can be either converted into energies using the Boltzmann distribution [8,25,26,38] or log-odds scores [40,41]. However, it has been shown that these two types of propensity are essentially the same [36]. In the second method, a potential function can be directly optimised in order to discriminate between native and near-native (decoy) structures [42]. This technique resembles machine learning, and has been applied in a variety of different ways, usually by maximising the discrimination between an average decoy and the native structure [43-46]. Either of the above two methods may be applied to any feature of the protein structure that can be parameterised [9]. In the current work, we focus on the statistical analysis of residue interaction propensities. Previously, a variety of different methods have been applied to derive empirical residue-residue interaction potentials, often yielding remarkably consistent results [27]. However, the physical basis of the empirically derived potentials remains ambiguous [47]. Specifically, it has been shown that protein structures are inconsistent with the assumptions that underlie the use of the Boltzmann distribution [28,48].

The major criticism of empirical residue-residue interaction potentials is that they ignore the protein/solvent boundary [27,28,48]. Consequently, there is an apparent attractive force between residues that co-segregate into the protein surface or core regions [28]. To address this, several groups have developed residue-specific environment potentials. These residue-specific environment potentials are usually correlated with hydrophobicity, measuring the extent to which each residue is buried in the protein core. In this way these single-body environment potentials capture information about the protein/solvent boundary. Such potentials have been combined with residue-residue interaction potentials: as a 'solvent correction factor' [49,50], as an ad-hoc repulsive term [38], and using a Bayesian framework to avoid over-counting [40].

The above combination of two-body, residue-residue interaction potentials with single-body, residue-specific environment potentials raises the question as to which type of potential is the most specific for the native protein structure. To address this question, we separated statistical residue interaction propensities into two different types of score: a two-body, residue-residue 'contact-type' score, and a single-body, residue 'contact-count' score.

These two types of score can be expected to capture qualitatively different kinds of residue interaction propensities. The resulting propensities can be understood in terms of biophysical properties of protein structure. For example, the contact-type score can encode the fact that hydrophobic residues tend to interact with other hydrophobic residues in preference to hydrophilic residues. In contrast, the contact-count score can encode the fact that bulky hydrophobic residues tend to have more residue-residue interactions than small hydrophilic residues.

Here we report a comparison of two-body, residue-residue 'contact-type' scores and single-body, residue 'contact-count' score, as described below.

## Results

**Figure 1:**
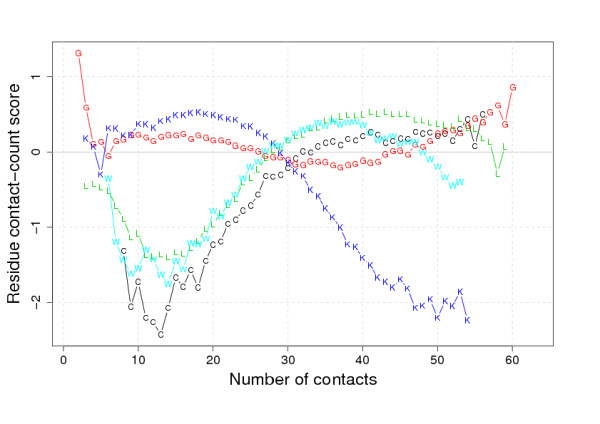
**An example of data from a contact-count scoring matrix**. An example of some data from one of the contact-count scoring matrices used in this work. The scores are defined as described in the Methods Section. In this matrix residue-residue contacts were defined using a 12 Å distance threshold between *C*_*β *_atoms without filtering for short-range interactions. Scores are shown for selected residues as trends across the range of observed 'number of contacts'. Missing values for a residue indicate cells of the matrix that were removed due to lack of data.

Two different types of residue interaction propensity are studied here, contact-type and contact-count. The 'two-body' residue contact-type propensities are based on the distinct amino acid types of a pair of contacting residues. The 'single-body' residue contact-count propensities are based on the discrete number of residue-residue contacts made by each distinct residue type. These two different interaction propensities are captured by the contact-type and contact-count scoring matrices, respectively. An example contact-type scoring matrix is given in Table 1, and the scores for some residues in an example contact-count scoring matrix are shown in Figure 1. The scores in these matrices reflect the observed residue interaction propensities in a set of native structures, and are defined in comparison to simple, random models of residue interaction. (For details see the Methods Section.)

**Table 1:** An example of data from a contact-type scoring matrix

	ALA	ARG	ASN	ASP	CYS	GLN	GLU	GLY	HIS	ILE	LEU	LYS	MET	PHE	PRO	SER	THR	TRP	TYR	VAL
ALA	0.27	-0.12	-0.19	-0.23	-0.06	-0.21	-0.39	0.11	-0.01	0.32	0.31	-0.42	0.00	0.26	-0.05	-0.05	0.05	0.12	0.19	0.35
ARG		-0.33	-0.38	-0.24	-0.30	-0.43	-0.36	-0.15	-0.22	-0.03	0.00	-0.72	-0.31	-0.01	-0.18	-0.27	-0.22	-0.10	-0.03	-0.01
ASN			-0.04	-0.30	-0.28	-0.34	-0.57	-0.09	-0.19	-0.02	-0.14	-0.47	-0.30	-0.01	-0.20	-0.13	-0.09	-0.10	0.02	-0.07
ASP				-0.44	-0.41	-0.48	-0.70	-0.18	-0.19	-0.14	-0.22	-0.43	-0.44	-0.15	-0.29	-0.28	-0.24	-0.19	-0.08	-0.15
CYS					0.67	-0.33	-0.63	-0.08	-0.02	0.15	0.12	-0.57	-0.06	0.19	-0.19	-0.13	-0.11	0.03	0.07	0.14
GLN						-0.36	-0.67	-0.26	-0.27	-0.10	-0.09	-0.64	-0.34	-0.08	-0.26	-0.28	-0.23	-0.13	-0.08	-0.11
GLU							-0.77	-0.45	-0.39	-0.23	-0.27	-0.53	-0.57	-0.27	-0.46	-0.51	-0.44	-0.38	-0.23	-0.25
GLY								0.16	0.02	0.16	0.08	-0.43	-0.10	0.15	-0.04	-0.01	0.04	0.08	0.14	0.19
HIS									0.16	0.09	0.07	-0.55	-0.11	0.16	-0.08	-0.05	-0.03	0.11	0.16	0.09
ILE										0.71	0.55	-0.13	0.24	0.55	-0.03	0.09	0.20	0.28	0.43	0.58
LEU											0.58	-0.26	0.15	0.49	-0.01	0.02	0.12	0.27	0.35	0.51
LYS												-0.63	-0.56	-0.23	-0.49	-0.47	-0.41	-0.45	-0.20	-0.22
MET													0.24	0.25	-0.23	-0.20	-0.11	0.09	0.14	0.16
PHE														0.60	0.04	0.10	0.16	0.39	0.47	0.48
PRO															-0.04	-0.15	-0.11	0.06	0.09	0.02
SER																-0.04	-0.03	0.01	0.07	0.07
THR																	0.06	0.05	0.13	0.21
TRP																		0.48	0.35	0.26
TYR																			0.42	0.37
VAL																				0.59

An example of one of the contact-type scoring matrices used in this work. The scores are defined as described in the Methods Section. In this matrix residue-residue contacts were defined using a 12 Å distance threshold between *C*_*β *_atoms and using a sequence separation filter to remove short-range interactions.

Both types of scoring matrix were constructed using several different definitions of residue-residue interaction. Three different structural criteria were used to define residue-residue interaction. Firstly, we tested the effect of the choice of atomic interaction site, representing each residue by either the *C*_*α *_atom or the *C*_*β *_atom, or both. Secondly, the contact distance threshold was varied between 6 and 20 Å in increments of 1 Å, giving a total of 15 different distance cutoffs. Thirdly, we applied a sequence separation filter, either considering all interactions or only the long-range interactions. Long range interactions were defined as interactions between residues that are more than 10 residues apart in the protein sequence [51,52]. The combination of these criteria gave a total of 3 (*C*_*α*_, *C*_*β *_or both) ×15 (distance cutoffs) ×2 (all or long range contacts) = 90 different residue-residue contact definitions.

The following three sections present the different contact-type and contact-count scoring matrices. First, the scoring matrices themselves are described, as they provide information on the nature of the captured residue interaction propensities. Second, the results of scoring native and 'fully-random' protein structures are presented. Third, the matrices are used to evaluate several sets of protein decoy structures.

In summary the results show that; i) the contact-count scores are much more specific than the contact type scores compared to random models of residue-residue interaction, ii) the *C*_*β*_-*C*_*β *_interaction captures the most specific residue interaction information compared to other atomic interaction sites, iii) both scores can identify 'unusual' proteins in the training set, iv) in contrast to point i, both scores perform equally well on the task of discriminating between decoy structures. The apparent contradiction between point i) and iv) will be returned to in the Discussion.

### The magnitude of the scoring matrices

**Figure 2:**
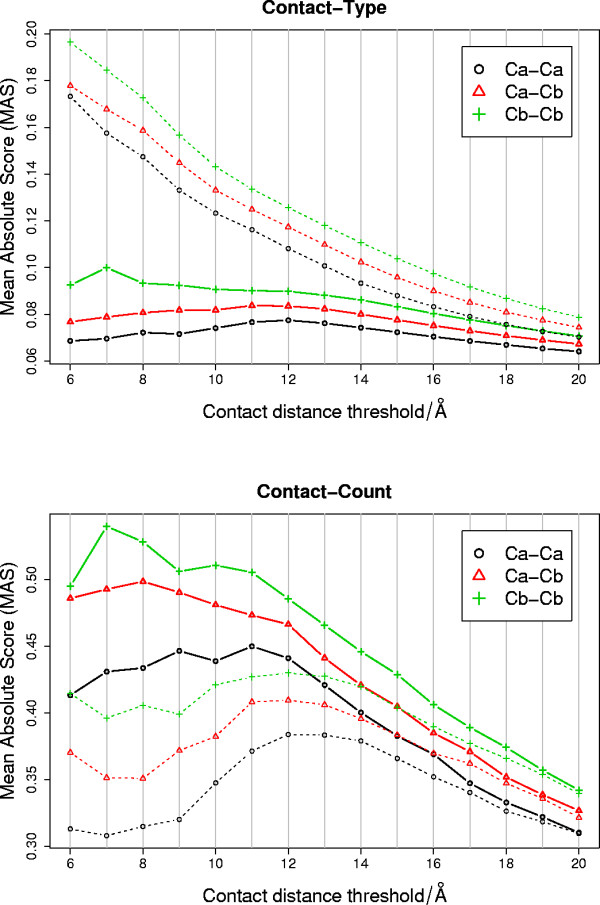
**Magnitude of the contact-type/count scoring matrices**. Each point gives the mean absolute score (*MAS*) of each cell in a particular residue interaction propensity scoring matrix. The different scoring matrices result from the different (given) residue-residue contact definitions used in matrix construction. The broken lines indicate the trend for the matrices with sequence separation filtering and the solid lines give the trend for the matrices without sequence separation filtering.

The 'mean absolute score' of a scoring matrix (*MAS*) was defined as the mean of the absolute value of the score in each cell of the matrix. The magnitude of *MAS *gives the degree to which the observed residue interaction propensities deviate from random. In other words, *MAS *measures the 'information content' of the observed interaction propensities encoded in the scoring matrix. The value of MAS would be equal to zero if residue interactions occurred at random, i.e. without any particular interaction propensities. The mean absolute score for each different contact-type and contact-count scoring matrix is shown in Figure 2, and are described in detail below.

#### Contact-type

The sequence separation threshold has the biggest effect on the mean absolute score (*MAS*) of the contact-type scoring matrices (Figure 2). Without sequence separation filtering, the contact-type scoring matrices tend to have smaller values of *MAS*. This clearly shows the effect of including the inherently non-specific short-range contacts in the scoring matrix. The scoring matrices that include short-range contacts are 'more random', with respect to the observed contacts encoded in the matrix. A similar effect is seen with increasing contact distance threshold.

#### Contact-count

**Figure 3:**
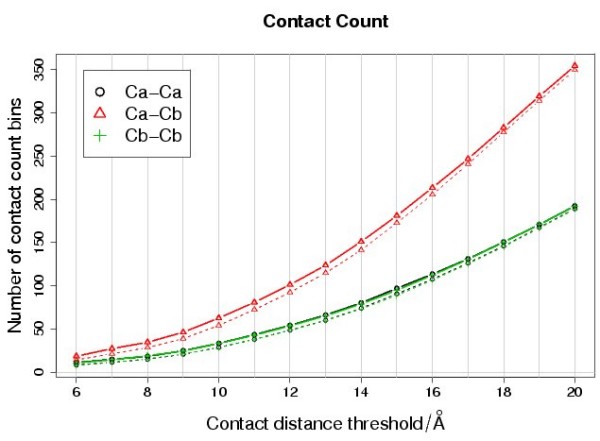
**Size of the contact-count scoring matrices**. Each point gives the number of different 'number of contact-bins' in the different contact-count scoring matrices. The broken lines indicate the trend for the matrices with sequence separation filtering and the solid lines give the trend for the matrices without sequence separation filtering (as described in the text). The plot shows how the 'number of contact-bins' varies with the contact definition.

The values of the 'mean absolute scores' (*MAS*) of the contact-count matrices are consistently larger than those of the contact-type matrices (Figure 2). The number of elements in the contact-count scoring matrix may vary with the residue-residue contact definition used (Figure 3). However, the value of *MAS *is comparable between the different types of scoring matrix because *MAS *is the mean absolute score over all elements in the matrix. The comparison suggests that the 'number of contacts per residue type' is consistently more informative than the 'residue-residue contact-type', given any of the residue-residue contact definitions used here. Unlike the contact-type matrices, the contact-count matrices appear consistently more informative when short-range contacts are included.

#### Summary

The 'Mean Absolute Score' shows that contact-count matrices are consistently more specific than the contact-type matrices. At all the distance thresholds, either with or without the sequence separation filtering, the values of *MAS *are largest for the *C*_*β*_-*C*_*β *_contacts, then the *C*_*α*_-*C*_*β *_contacts, then the *C*_*α*_-*C*_*α *_contacts. This shows that the *C*_*β *_atom captures the specific side chain interactions more accurately than either of the other two definitions.

### Scoring native protein structures

Each of the scoring matrices was derived from a data set of 3, 070 monomers (see the Methods Section for details). As a simple test, each of the native proteins was scored using the contact-type and contact-count scoring matrices which had the highest value of *MAS *(as described above). Using either the contact-type or the contact-count scoring matrices, there were some proteins that scored significantly worse than average. Examination of the 130 worst cases showed that they were caused by a few anomalies and annotation errors.

There were 86 very small proteins and protein fragments. These included, for example, the structure of single *C*_*α*_-helices and extended, coiled-coil proteins. There were 25 membrane associated proteins, including alpha helical and beta-barrel lipoproteins. There were 12 proteins that adopted an extended conformation in complex with either DNA or several large ligand groups. Another four structures were found to be *C*_*α *_only models, containing only the backbone and no side-chain information.

Interestingly, in this group we found 3 structures of protein subunits from oligomeric proteins. These cases were incorrectly annotated monomers in the data set. These subunits appear 'non-native' because they would make many additional residue contacts in the native oligomer. For this reason the artificially isolated subunit is effectively 'non-native' and scored badly as a result.

An examination of 130 randomly selected proteins from the data set showed only a few protein fragments and DNA binding proteins. There were several proteins found binding large ligand groups, but the relative extent of the ligand was small compared to the cases found above. There were no trans-membrane structures found in the random sample.

For the above reasons, these 130 cases were removed from the data set giving a total of 2, 940 monomers. The matrices were re-calculated over this new data set for use in the following sections.

### Scoring 'decoy' structures

In this section we describe a realistic benchmark of score performance [53] using several standard sets of 'near-native' protein decoys [54]. Here the scores are used to evaluate the decoys with reference to the *C*_*α *_RMSD of the decoy to its corresponding native structure. The *C*_*α *_RMSD is used as an independent measure of decoy quality in order to evaluate the various scores.

#### Description of the decoy sets used

Nine different sets of decoys were used in the current work. The structures of the decoys were taken from the Decoys-R-us database [54]. Each decoy set uses a particular method to generate several 'near-native' protein structures using a given native protein structure. Some additional information for the different decoy sets is given in Table 2. The different methods include: energy minimisation (lmds and vhp-mcmd), homology modelling (hg-structal, ig-structal, and ig-structal-hires), systematic randomisation with subsequent filtering (4-state-reduced and lattice-ssfit), *ab-initio *(semfold) and *de-novo *methods (fisa and fisa-casp3).

**Table 2:** Summary of the nine different decoys sets from decoys-R-us

	Decoys					Range of RMSD		
DataSet	NoP	NoR	MLen	NoD	MD	Min.	Median	Max.

vhp-mcmd	1	36	33	6256	6256	0.5	7.3	12.8
hg-structal	29	4338	141	870	30	0.5	3.0	30.3
4-state-reduced	7	448	60	4659	666	0.8	5.5	9.4
ig-structal-hires	20	4548	224	400	20	0.7	2.1	6.4
ig-structal	61	13893	224	3720	61	0.7	2.0	6.8
fisa	4	241	52	2003	501	2.8	7.4	14.1
fisa-casp3	4	368	82	5995	1499	3.6	11.6	20.9
lmds	10	534	48	4346	435	2.4	7.8	13.5
semfold	6	440	68	32718	13037	0.1	10.7	15.1
lattice-ssfit	8	565	67	8288	2000	4.7	9.8	15.6

Totals	150	25411		69255				

The nine different sets of decoys taken from the Decoys-R-Us database [54]. For each decoy set, the number of proteins (NoP) in the set is given, along with the total number of residues (NoR) and the mean length of the proteins (MLen). The total (NoD) and mean (MD) number of decoys per-protein is also given. Within each decoy set, the range of RMSD values over all the decoys in the set are indicated, along with the median of that distribution.

#### The relationship between decoy 'quality' and score

When assessing the relationship between decoy 'quality' and the residue interaction propensity score, several different measures of score performance are important [53]. Here we apply two different measures of score performance, collected from the decoys as described below. The first measure is the Spearman rank correlation coefficient (S). The value of S shows whether the interaction propensity score can accurately discriminate between decoys of varying quality. The second measure is the Z-score of the native structure compared to the decoys with respect to interaction propensity score (Z). A large and positive Z indicates a clear discrimination of the native conformation from that of the decoys using the interaction propensity score.

The nine different decoy sets were analysed separately, and each decoy was scored using the different residue contact-type and contact-count scoring matrices as described above. The two different measures of score performance described above (S and Z) were calculated for each protein. For each decoy set we always report the mean value of S and Z over all the proteins in the set, given a particular residue-residue contact definition. In the following sections we refer to the 'best' score for a decoy set as the contact definition that had the best mean performance (on S or Z) over all the proteins in the set.

##### The best values of S per decoy set

Focusing only on the best performing scoring matrices, we saw considerable variation between decoy sets. The best values of S per decoy set varied between 0 and 0.7 (Table 3). Four of the nine decoy sets showed very little correlation (S below 0.30). Another four had some correlation (S between 0.3 and 0.6) and only two of the nine showed a reasonable correlation between score and quality (S above 0.6).

**Table 3:** The best results for each of the nine different decoy sets

	Type		Count	
Decoy Data Set	Spearman	Z-Score	Spearman	Z-Score

vhp-mcmd	-0.69	1.87	-0.57	2.92
hg-structal	-0.57	1.61	-0.68	1.44
4-state-reduced	-0.52	2.51	-0.45	1.94
ig-structalhires	-0.41	1.59	-0.38	1.62
ig-structal	-0.33	1.22	-0.28	1.59
fisa	-0.30	1.36	-0.36	2.59
fisa-casp3	-0.18	0.03	-0.25	1.43
lmds	-0.13	0.86	-0.12	1.72
semfold	-0.09	-	-0.09	-
lattice-ssfit	-0.03	3.76	-0.09	3.36

Mean value	-0.33	1.65	-0.33	2.07

Table of the best results for each of the nine different decoy sets. The values given are the mean over each protein in the decoy set, and are the best obtained using any of the different residue interaction propensity scores.

The best scoring contact-type and contact-count scoring matrices have very similar performance over the nine different decoy sets. The nine different contact-type and contact-count S values in Table 3 have a Spearman rank correlation coefficient of 0.95. This clearly shows that the contact-type and contact-count scores have equivalent performance on the discrimination task. In all cases, a strong (or weak) correlation using the contact-type scores implies a strong (or weak) correlation using the contact-count scores.

##### The best values of Z per decoy set

The best values of Z for contact-type and contact-count are less strongly correlated, having a Spearman rank correlation coefficient of 0.7 (Table 3). In addition, the best Z do not correlate well with S. For one case in particular (lattice-ssfit) weak S is accompanied by a large Z (Table 3).

In general, the best contact-type scoring matrices have worse Z than the best contact-count scoring matrices (Table 3). In one case in particular (fisa-casp3), the best contact-type Z is very small (0) and the contact-count Z is moderate (1.4). However, the difference in the Z between the two different score types is not significant (p = 0.1, df = 8).

In the above two paragraphs we described the relative performance of the best contact-type and contact-count scoring matrices. The important question of which residue-residue contact definitions give the 'best' performance of S and Z is addressed in the following paragraph.

##### Choosing a specific residue-residue contact definition

The choice of a specific residue-residue contact definition can have a large and significant effect on the results of the scoring matrices. The performance can vary, not just between count and type scoring matrices, but also between different decoy sets. For example, the best S for the contact-count score occurs at *C*_*β*_-*C*_*β *_8 Å without sequence separation for the 4-state-reduced decoy set, but at *C*_*β*_-*C*_*β *_14 Å without sequence separation for the fisa decoy set. Using these contact definitions the values of S are 0.45 and 0.35 for the two decoy sets, respectively. Exchanging the contact definitions in these two cases, S falls to 0.40 and 0.25, respectively.

**Figure 4:**
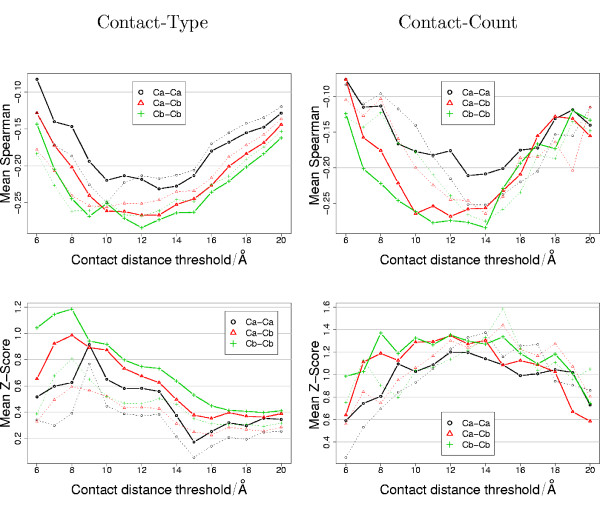
**Mean performance**. The mean value of S (upper) and Z (lower) for the contact-type (left) and contact-count (right) scoring matrices over the nine different decoy sets. The means were calculated as the mean of the mean value per decoy set, rather than the mean over the total set of proteins. The broken lines indicate the trend for the matrices with sequence separation filtering and the solid lines give the trend for the matrices without sequence separation filtering.

The ultimate aim of a scoring function is to rank near-native protein decoys according to their similarity to the native structure. The performance of the scoring function on this task should be independent of the method used to generate the decoys. For this reason, it is informative to look at the overall performance of each different scoring matrix across all the different decoy sets. The mean values of S and Z for each different scoring matrix over each decoy set are presented in Figure 4.

The best performance of the contact-type scoring matrices is obtained by defining residue-residue contact using *C*_*β*_-*C*_*β *_atoms with a distance threshold of 12 Å. This is obtained without sequence separation filtering, including short-range contacts. The best performance of the contact-count scoring matrices occurs at slightly longer distance threshold of 14 Å (Figure 4). Overall, the contact-count and contact-type matrices show a similar pattern of performance across different residue-residue contact definitions. The best Z are generally found using the contact-count scoring matrices. Both types of scoring matrix have a maximum in Z when using *C*_*β*_-*C*_*β *_atoms with a distance threshold of 8 Å. In addition, the Z of the contact-count scoring matrices is also high between 10 and 16 Å

## Discussion

Early work on single-body 'residue environment' potentials was very promising [2,3,55-63]. However, the effectiveness of these potentials has never been directly compared to two-body 'residue pair' potentials in detail. Here we do not try to combine the two different types of residue interaction propensity score, but rather to assess their independent contribution to scoring protein structures. The objective is to examine how much information is stored in the two types of measure and to compare their performance on the realistic task of ranking a set of decoy structures.

### The magnitude of the scoring matrices

To address the question of which type of residue contact propensity score contains the most specific information about protein structure, we assessed the mean absolute score (MAS) of the cells in the different scoring matrices. The score in each cell measures the strength of a certain residue contact propensity. In this sense, magnitude of the MAS gives the degree of 'non-randomness' or information content of the given residue contact propensity. The MAS suggests that, whatever the residue-residue contact definition used, the 'single-body' residue contact-count propensities were stronger or more informative than the 'two-body' residue contact-type propensities.

As the residue-residue contact definition was changed, we observed changes in *MAS *that were consistent with previous observations [64]. The most informative contact-type and contact-count matrices were obtained using *C*_*β*_-*C*_*β *_contacts at 6 Å without sequence separation filtering and using *C*_*β*_-*C*_*β *_at 7 Å with sequence separation filtering, respectively. However, the pattern of change in *MAS *that occurred as a consequence of changing residue-residue contact definition were not seen in the score performance on the task of scoring protein decoys.

#### Scoring native protein structures

Scoring the data set of 3, 070 native proteins highlighted some problematic structures. Some of the worst scoring proteins in this set when using either the contact-type or the contact-count scoring matrices were all found to be membrane proteins. It is not surprising that the residue contact propensities derived from a data set of mostly globular proteins are not generally the same as the propensities seen in membrane proteins.

Further down the list of the worst scoring native proteins, we find some protein subunits of oligomeric proteins that were incorrectly annotated monomers. These subunits appear 'non-native' because they would make many additional residue-residue contacts in the native oligomer. The artificially isolated subunit is effectively 'non-native'.

#### Ranking near-native protein decoys

Firstly, we observed that the decoys in some sets cannot be successfully ranked by either the contact-type or the contact-count scores. These sets of decoys are all considered equally 'native' (or equally 'non-native') by the residue contact propensity scores, despite having a range of different RMSD values to the native structure [54]. We observed that these decoy sets lacked decoys in the range of 1 to 5 Å RMSD, having less than 25% of the decoys below 5 Å. This observation suggests that the scores might perform better on decoys that are closer to native.

**Figure 5:**
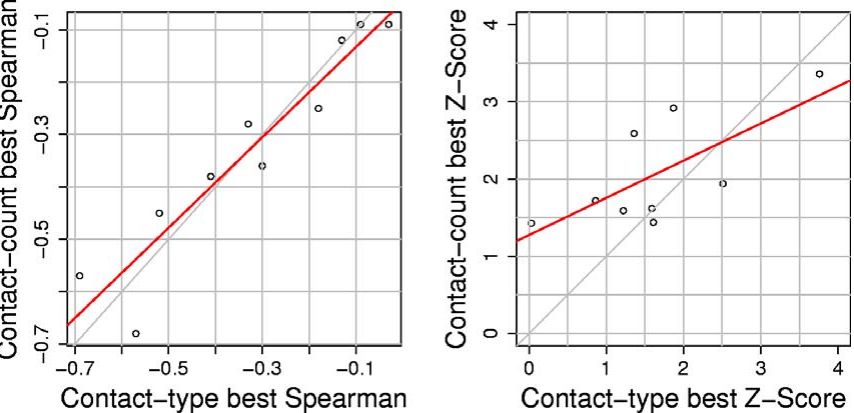
**Performance of the two different types of score**. The best values of S and Z for the contact-type and contact-count scoring matrices. Each point represents one of the nine decoy sets studied in this work, encompassing the scores from 150 proteins and 244,794 decoys.

Secondly, and perhaps more importantly, we observe that the two different kinds of score perform equally well on the different decoy sets (Figure 5). The contact-type and contact-count performance in terms of both rank correlation coefficient (S) or Z-score (Z) are both highly correlated. The correlation of the best performance is 0.97 for S and 0.67 for Z.

Finally, we observed that the specific residue-residue contact definition that gave the best performance varied between the different decoy sets studied. However, similar trends in performance were observed at any given residue-residue contact definition across all sets.

Several other groups have reported good performance on similar discrimination tasks using single-body residue burial terms. For example, in Godzik et. al. 1992 [60] it was reported that, in most cases, a burial term alone is a sufficient indicator of the native sequence compared to two- and three-body residue interaction terms. A Bayesian scoring function developed in Simons et. al. 1999 [9] suggested that residue burial scores have comparable performance to residue contact scores. Similarly, in Zhou et. al. 2004 [65] the authors concluded that the the residues solvent accessible surface area appears to be the most important among several different single-body terms tested. In addition, several groups have used a similar definition of residue contact-count as an approximation for burial [48,50,65-67]).

The current work suggests that counting contacts between *C*_*β *_atoms using a distance threshold around 12 Å provides the most discriminative single body residue contact-count score (Figure 4). Similar observations have also been confirmed in the literature. For example, in Karchin et. al. 2004 [68] the best results were obtained with a 14 Å contact definition between *C*_*β *_atoms.

However, in a number of studies a distance threshold of 9 Å between *C*_*β *_atoms was used to count contacts [64,69]. In one such study, it was stated that the 9 Å distance threshold used resulted in a slightly better performance than other cutoffs tested [69]. This difference may result from the specific count normalisation procedure applied in that work.

Only two different sequence separation filters were assessed in detail in this work, considering either all interactions or only the long-range interactions. Long range interactions were defined as interactions between residues that are more than 10 residues apart in the protein sequence. Results collected using alternative sequence separation thresholds of 5, 8 or 12 showed gave very little change in the scores collected. Using a sequence separation threshold of 2 showed scores that were roughly in between those of 0 and 10. It important to note that when scoring near-native decoys, sequence-separation filtering has very little effect on the performance of the score, as all decoys and the native protein have the same primary sequence.

#### Cooperativity in protein folding

It has long been suggested that pairwise potentials may not capture the inherent cooperativity of protein folding (for example see [14,70]). Here we have presented results for the effectiveness of the contact-count score, suggesting that indeed higher order interactions are indeed important in protein structure. For example, it has been shown that contact-count can be estimated from a four-body residue-residue interaction potential [71]. However, the performance of such a four-body potential, assessed using the (SNAPP) score [72], is not significantly better than an equivalent two-body potential [73]. Despite this observation, four-body potentials are becoming much more commonly used as a way to better capture the cooperativity of protein interactions [74].

#### Future directions

The work presented here represents a basic comparison of contact-type and contact-count scores. There are several ways in which this basic work should be extended. However, it is important to note that the two scores compared in this work are far from optimal. It is known that distance dependent all-atom scores are more effective at discriminating between native and non-native protein structures [48,69]. Developing the current work along these lines will be an important task for the future. In particular, it remains to be seen if the findings presented here at the residue level are consistent with observations at the atomic level.

We did not directly compare the statistical potentials derived in this work to similar potentials described by other authors in the literature. To extend the analysis presented here, our potentials should be compared directly with those in the literature (for a good example of this type of comparison see [75]) Additionally, a comparison of the important amino acid properties such as hydrophobicity and electrostatics should be performed.

In this work we did not address combinations of the two scores. The two types of potential studied perform equivalently, suggesting that they are based on a similar underlying principle. However, if a combination of scores improves the overall performance this would show that the scores carry different information. Although that an ideal scoring function should work in all possible cases, correlation between RMSD and score is usually only significant for RMSD below about 3 Å [76]. For this reason it would be useful to compare the scoring functions on decoys within specific ranges of RMSD from the native.

## Conclusion

In this work we assessed the independent contribution of two different types of residue contact propensity to scoring protein structures. The main finding is that the contact-type and contact-count scores showed equivalent overall performance in the task of ranking protein decoys. Although the two different score types perform equivalently, the ability to automatically predict the number of contacts made by a residue [68,77,78] allows for a greater range of applications. In addition, a single-body term is amenable to an efficient dynamic programming method for alignment optimisation [3,65,79].

The work presented here represents our preliminary investigation of a multi-body potential for evaluation of protein structure. In future it should be possible to combine the contact-type and contact-count scores to better take into account the inherent cooperativity of protein folding.

## Methods

### The data set of native proteins and protein decoys

#### The non-redundant data set of monomers

The scoring matrices were derived from a non-redundant set of high-quality protein monomers. This set of 3, 070 proteins was selected using the following protocol. Only the monomers from the BioUnit section of the Protein Data Bank (PDB) [80,81] were selected, excluding putative structures of dimers, trimers, and the other multi-subunit proteins. The resulting monomeric proteins were further filtered by size, having more than 20 amino acids, and by resolution, being better than 3 Å. Finally, the chains were made non-redundant at 30% sequence identity using BLASTClust [82]. The resulting set of 3, 070 monomers was used throughout this analysis.

#### The data set of 'near-native' protein decoys

Nine sets of protein decoys were taken from the Decoys R us database [54]. In total this data set included 244, 794 decoys derived from 150 native proteins.

### Constructing the scoring matrices

#### Contact-Type

Given the fraction of residues of type *x *and of type *y *(*P*_*x *_and *P*_*y*_), the probability of randomly observing a contact of type *xy *is,

(1) *P*_*xy *_= *P*_*x*_·*P*_*y*_

where *P*_*xy *_is the probability of a 'random' contact of type *xy *(for example see Table 1). This formula is obtained by assuming that contacts are made between randomly selected pairs of residues, assuming statistical independence. In this way, we make no assumptions about the distribution of contacts within the protein, such as the distribution of the number of contacts per residue.

The observed and expected probabilities of a contact of type *xy *can be combined into a score using the log-odds ratio;

(2) $$S_{xy}=\log\left(\frac{P_{xy}^{obs}}{P_{xy}^{exp}}\right)$$

The magnitude of the score *S*_*xy *_gives a measure of how 'non-randomly' the pair *xy *occurs. The score is positive when *xy *is observed more often than expected and negative when *xy *is observed less often than expected.

#### Contact-Count

The contact-count scoring matrix is created in a similar way to the contact-type scoring matrix. However, instead of using the frequency *P*_*xy *_to denote the probability of a residue-residue contact between residue type *x *and type *y*, we use *P*_*xn *_to denote the probability of a residue of type *x *having exactly *n *residue-residue contacts. The probability *P*_*xn *_is defined as,

(3) *P*_*xn *_= *N*_*xn*_/*N*_*n*_

where *N*_*xn *_is the observed number of residues of type *x *having exactly *n *contacts and *N*_*n *_is the total number of residues with exactly *n *contacts (for example see Figure 1). The values of *n *were taken from those observed over all residues. The 'random' value of *P*_*xn *_is simply taken to be equal to *P*_*x*_, the fraction of residues of type *x*. Using this value assumes that there is no particular effect on the overall amino acid composition when filtered by a given number of contacts. Again the observed and expected probabilities can be combined as in Equation 2. Using this method, the magnitude of the score *S*_*xn *_can be easily interpreted as a measure of 'compositional bias' given a certain number of residue-residue contacts.

#### Undersampling

Certain residue-residue contact definitions could lead to low counts in the scoring matrices. For example, if the overall number of observed residue-residue contacts is low, certain contact-types may become rare. Similarly, if the number of different contact-counts spans a wide range, instances of a given residue type with a given contact-count may become rare. To address this issue of undersampling, if a cell of a scoring matrix was based on fewer than 5 observed or expected counts, that score was discarded. Those specific classes of contact were therefore ignored when scoring protein structures, being neither penalised nor rewarded.

Although a threshold of 5 observed or expected counts was used to filter undersampled classes in the results presented here, it should be noted that both the contact-type and contact-count scores appear very robust, showing only small changes in MAS when discarding cells with fewer than 50 observed or expected counts. The complete set of counts and scores for the contact-type and contact-count scoring matrices are included as additional files (see Additional file 1).

## Authors' contributions

DB conceived and designed the study, performed the statistical analysis and drafted the manuscript. IF helped design the study, helped write software for acquisition of data and helped draft the manuscript. HS helped write software for acquisition of data and helped draft the manuscript. JD wrote the software for acquisition of data. ML participated in design and coordination of the study and helped to draft the manuscript. All authors read and approved the final manuscript.

## Supplementary Material

Additional file 1 **The complete set of counts and scores for the contact-type and contact-count scoring matrices**. The zipped tar archive contains four directories containing all the residue contact data that was used in the analysis, along with the resulting scores. All data within the archive is provided as tab-delimited flat-files in text format. Click here for file
